# Effects of glycerol hyperhidration on the running economy of long-distance runners: a randomized crossover clinical trial

**DOI:** 10.3389/fnut.2025.1630462

**Published:** 2025-08-13

**Authors:** Carlos Abraham Herrera-Amante, Gustavo García-Zepeda, Carlos Eduardo García-Zepeda, Rodrigo Yáñez-Sepúlveda, Vicente Javier Clemente-Suárez, José Francisco López-Gil, César Octavio Ramos-García

**Affiliations:** ^1^Nutritional Assessment and Nutritional Care Laboratory (LECEN), Division of Health Sciences, Tonalá University Center, University of Guadalajara, Tonalá, Mexico; ^2^Research Division, Ibero-American Institute of Sports Sciences and Human Movement (IICDEM), Guadalajara, Mexico; ^3^Ibero-American Network of Researchers in Applied Anthropometry (RIBA^2^), Almería, Spain; ^4^Faculty of Education and Social Sciences, Universidad Andres Bello (UNAB), Viña del Mar, Chile; ^5^Faculty of Sports Sciences, Universidad Europea de Madrid (UE), Madrid, Spain; ^6^Grupo de Investigación en Cultura, Educación y Sociedad, Universidad de la Costa, Barranquilla, Colombia; ^7^School of Medicine, Universidad Espíritu Santo, Samborondón, Ecuador; ^8^Vicerrectoría de Investigación y Postgrado, Universidad de Los Lagos, Osorno, Chile

**Keywords:** glycerol, athletic performance, sports nutritional sciences, thermoregulation, running, performance-enhancing substances

## Abstract

**Background:**

Endurance athletes experience significant water loss during exercise, which can impair performance and increase the risk of dehydration. Glycerol hyperhydration has been explored as a strategy to enhance pre-exercise hydration, particularly when fluid intake opportunities are limited. This study aimed to evaluate the effects of glycerol hyperhydration on running economy (RE) in trained runners.

**Methods:**

A randomized crossover clinical trial was conducted with 30 trained runners (15 men, 15 women) across three sessions. In the first session, peak oxygen uptake (VO_₂_peak) was determined to establish individualized running speeds for the subsequent tests. In the second and third sessions, participants performed submaximal running tests under two conditions: euhydration (control) and glycerol-induced hyperhydration. The glycerol protocol consisted of ingesting 1.2 g/kg body mass of glycerol diluted in 22 mL/kg of water, 120 min before exercise. The assessed variables included caloric cost unit (CCU), oxygen cost unit (OCU), heart rate (HR), body temperature (BT), and rating of perceived exertion (RPE). Effect sizes were calculated using standardized mean differences (Cohen’s *d*).

**Results:**

Glycerol supplementation significantly improved running economy, reducing CCU (*p* = 0.025, d = 0.43), OCU (*p* = 0.011, r = 0.46), HR (*p* = 0.029, d = 0.42), and RPE (*p* = 0.003, d = 0.60). Although BT showed a slight decrease (*p* = 0.053, d = 0.37), it did not reach statistical significance, suggesting a trend toward improved thermoregulation.

**Conclusion:**

These findings indicate that glycerol supplementation enhances key metabolic and physiological factors associated with running economy, including CCU, OCU, cardiovascular responses, and perceived exertion in trained runners.

**Clinical trial registration:**

https://clinicaltrials.gov, identifier: NCT06818253.

## 1 Introduction

Glycerol, also known as 1,2,3-propanetriol or glycerin, is a three-carbon lipid-soluble molecule (C_₃_H_₈_O_₃_) that appears as a colorless, viscous, and hygroscopic liquid at room temperature that is capable of absorbing moisture from the environment (1). After ingestion, glycerol is absorbed in the small intestine, where it increases osmotic pressure and plasma volume, promoting fluid retention in the nephron. This leads to a decrease in urine volume (2), which helps to increase the body’s hydration status (3, 4).

Owing to its properties, glycerol supplementation has been proposed as a promising ergogenic aid and a hyperhydrating agent to enhance athletic performance. Glycerol-induced hyperhydration improves fluid retention, contributing to an effective hyperhydration status (5, 6). When added to water or sports drinks before exercise, glycerol has several benefits, including faster heart rate recovery (7), enhanced body temperature regulation and reduced rectal temperature, particularly in hot and humid environments (8–11); reduced perceived exertion (12); and improved hydration levels (13, 14).

In endurance sports, maintaining an adequate euhydration state is a key factor for performance and is especially challenging. Fluid loss during exercise, averaging 1.28 L/h in endurance athletes (15), often exceeds 2% of their body mass, negatively affecting thermoregulation, cardiovascular function, and, consequently, physical performance (16). These losses are exacerbated by high physical demands, adverse weather conditions, and limited opportunities to replenish fluids during competitions.

Thus, glycerol supplementation represents a promising strategy to mitigate these adverse effects. However, findings on its effectiveness have been inconsistent, likely owing to study design limitations, such as intake protocols (e.g., differences in supplementation timing prior to performance tests) or insufficient fluid intake volumes combined with glycerol, leading to considerable variability in body mass fluctuations before and after supplementation. These discrepancies highlight the need for more robust research controlling these variables.

Pioneering studies reported significant improvements in fluid retention, thermoregulation, and physical endurance with doses of 1.2 g/kg glycerol combined with 20–25 mL/kg fluid or sports drink compared with hyperhydration with water alone (3, 17). However, subsequent research failed to achieve consistent performance results (10, 18–20). These findings underscore the need to investigate the effects of glycerol under controlled conditions and to standardize supplementation protocols across studies.

Despite its reported efficacy, glycerol was included in the World Anti-Doping Agency (WADA) list of prohibited substances in 2010 because of its potential use as a masking agent for banned substances in doping tests (21). However, with the introduction of the biological passport and advancements in detection methods, glycerol was removed from this list in 2017, reinstating its acceptance for use in sports (22).

Currently, glycerol is recognized as a safe and effective ergogenic aid (23), included in recent frameworks such as that of the Australian Institute of Sport (24). However, only a few studies have evaluated its impact on running economy (RE), defined as the amount of oxygen and calories consumed to maintain a constant running speed (25, 26). For example, Beis et al. (27) reported no significant effects of glycerol supplementation on RE, although they observed increases in body mass due to fluid retention. These findings suggest limited and inconclusive evidence regarding glycerol’s impact on RE, highlighting the need for further investigation under controlled experimental conditions.

Therefore, this study aimed to evaluate the effects of hyperhydration with glycerol, in combination with water, on running economy in long-distance runners compared with a euhydration state. The hypothesis is that hyperhydration with glycerol might negatively impact at least one RE variable because of the significant increase in body mass after supplementation.

## 2 Materials and methods

### 2.1 Study design

The study was designed as an experimental, quantitative, randomized, counterbalanced, crossover clinical trial consisting of three intervention sessions conducted in a controlled environment. This crossover design minimized interindividual variability by using each participant as their own control to evaluate two conditions: (i) hyperhydration with glycerol (Gly) and (ii) euhydration without glycerol (No-Gly). The order of these conditions was randomized to control for order and learning effects. The primary variables assessed were peak oxygen consumption (VO_₂_peak), running economy (RE) calculated using the oxygen cost unit (OCU) and the caloric cost unit (CCU), body temperature (BT), Borg’s rating of perceived exertion (RPE), and hydration status. Experimental trials (Gly and No-Gly) were conducted 24 h apart and at the same time of day (±30 min) to control for circadian variation. Nutritional intake in the 24 h prior to each test was replicated based on a standardized dietary record, and participants were instructed to avoid vigorous exercise between sessions. This trial was conducted in accordance with the Consolidated Standards of Reporting Trials (CONSORT), ensuring quality and transparency in the reporting of results (28).

### 2.2 Setting

The assessments were conducted between August 23, 2021, and April 15, 2022, at the Exercise Physiology and Nutritional Status Assessment Laboratory of the Universidad Contemporánea de las Américas (UNICLA), located in Morelia, Michoacán, Mexico. This laboratory offers controlled conditions and specialized equipment for exercise physiology research. The study adhered to the ethical principles outlined in the Declaration of Helsinki and its latest amendments, as well as international guidelines for Good Clinical Practice (29). The protocol was approved by the Interinstitutional Ethics Committee of the Iberoamerican Institute of Sports Sciences and Human Movement (Code: CEI-IICDEM-01-08-2021), and it was registered at ClinicalTrials.gov (Identifier: NCT06818253).

### 2.3 Participants

Competitive long-distance runners were recruited through an open call in athletics clubs in Morelia, Michoacán, Mexico. The inclusion criteria were as follows: (i) competitive runners with a minimum pace of 4.2 min/km, (ii) aged between 18 and 35 years, (iii) apparently healthy athletes, and (iv) signed informed consent. The exclusion criteria included (i) the presence of comorbidities that could affect the study variables (e.g., hypertension, hyperthyroidism, or heart disease), (ii) the use of medications or stimulants (e.g., caffeine or diuretics) 48 h prior to testing, and (iii) pregnancy. The elimination criteria were as follows: (i) injuries or illnesses during the study, (ii) urine specific gravity outside the allowed range (1.018–1.024), (iii) a resting heart rate exceeding 5 bpm compared with the initial evaluation, and (iv) inability to complete all three sessions of the protocol.

### 2.4 Interventions

**Figure 1:**
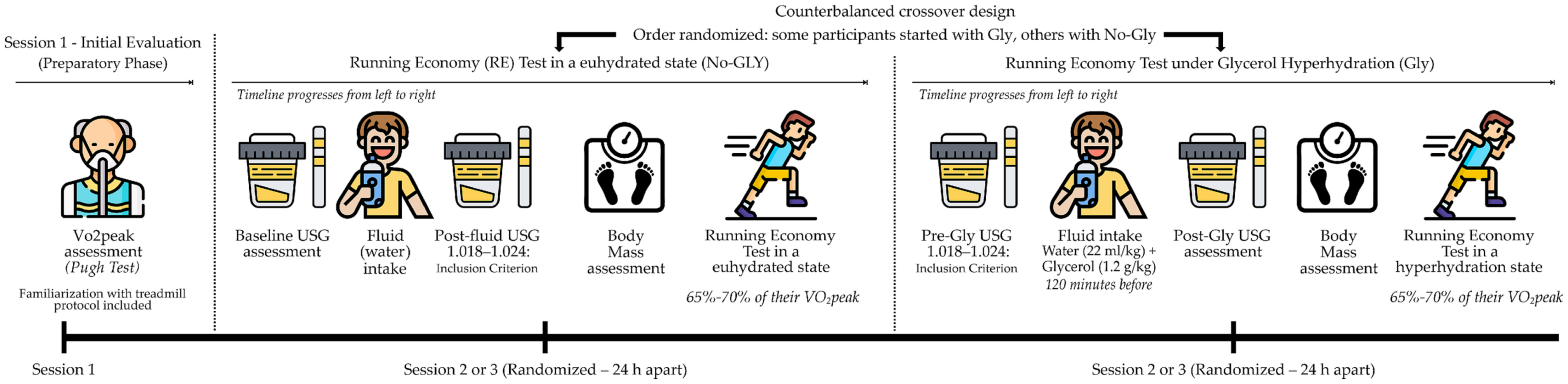
Overview of the experimental protocol. Session 1 involved VO_₂_peak testing (Pugh test) and treadmill familiarization. Participants then completed two experimental trials—No-Gly (euhydration) and Gly (glycerol-induced hyperhydration)—in a randomized, counterbalanced order, with each trial performed 24 h apart. Hydration status was assessed twice during each trial: upon arrival (baseline hydration) and after fluid intake, to verify euhydration (USG 1.018–1.024) in No-Gly or hyperhydration in Gly, before starting the running economy test at 65–70% VO_₂_peak.

The study was conducted in three sessions (Figure 1):

#### 2.4.1 Session 1—initial evaluation (preparatory phase)

During this session, participants signed an informed consent form and underwent clinical and nutritional history assessments. VO_₂_peak was measured using an incremental treadmill test (Pugh test), which started at 6 km/h and increased by 2 km/h every 3 min, with a constant 1% incline, until volitional exhaustion (30). Vital signs—including body temperature, blood pressure, and resting heart rate—were recorded, along with anthropometric variables such as body mass and stretch stature. Participants also familiarized themselves with the treadmill protocol that would be used during the running economy tests.

#### 2.4.2 Sessions 2 and 3—experimental trials (No-Gly and Gly conditions)

Following the initial evaluation, participants completed two running economy (RE) tests under different hydration conditions: a euhydrated state (No-Gly) and glycerol-induced hyperhydration (Gly). The order of the trials was randomized and counterbalanced among participants to control for order and learning effects.

Experimental trials were conducted 24 h apart, and each session was scheduled at the same time of day (±30 min) to minimize circadian variation. This short interval was chosen to reduce the potential for training adaptations and ensure comparable physiological conditions between trials.

To control for nutritional status, participants were instructed to replicate their food intake during the 24 h prior to each test, using a standardized dietary record provided by the research team. Furthermore, they were advised to avoid any vigorous physical activity between sessions to prevent interference with the physiological variables under study, particularly running economy.

For both trials, participants arrived at the laboratory 150 min before the RE test. In the No-Gly condition, they ingested water (150–200 mL every 15 min) to achieve a euhydrated state. Hydration status was verified by measuring urine specific gravity (USG) after completing the hydration protocol, with an acceptable range of 1.018–1.024 (31), prior to initiating the running economy test. In the Gly condition, participants followed the same hydration protocol to achieve euhydration, which was verified by USG (1.018–1.024); once this criterion was met, they consumed water (22 mL/kg) combined with glycerol (1.2 g/kg). The full volume was ingested over 30 min, followed by a 120-min rest period prior to the RE test.

In both sessions, participants ran for 15 min on a treadmill at a speed corresponding to 65–70% of their VO_₂_peak, as determined in Session 1. Measurements were identical across conditions, including the caloric cost unit (CCU), oxygen cost unit (OCU), body temperature (BT), and rating of perceived exertion (RPE) via the Borg scale.

RE calculation:

- The CCU was calculated via the following equation (25):

$$\mathrm{CCU}\;(\text{Kcal}\times {\mathrm{kg}}^{-1}\times {\mathrm{km}}^{-1})=\frac{VO_{2}\;\mathrm{ABS}\times (1.2256\times \mathrm{RER}+3.8204)}{S\times \mathrm{BM}}$$

where VO_2_ is the oxygen consumption rate (ml/min); RER is the respiratory exchange ratio; S is the speed (m/min); and BM is the body mass (kg).

- The OCU was calculated via the following equation (26):
$$\mathrm{OCU}\;(\mathrm{mL}\times {\mathrm{kg}}^{-1}\times {\mathrm{km}}^{-1})=(\frac{60}{S})\times VO_{2}\;\mathrm{REL}$$

where S is the speed (km/h) and VO_2_ is the oxygen consumption (ml·kg^−1^·min^−1^).

All tests were conducted under controlled thermoneutral conditions (20–25°C; 50–60% relative humidity), which were monitored using a thermal stress meter (EXTECH® HT30) for environmental parameters. Body temperature was measured using an infrared thermometer (Berrcom® JXB-178). All instruments and equipment, including digital scales (SECA® 803), digital refractometers (ATAGO® PAL-10S), and an indirect calorimeter (KORR® Cardiocoach Plus—model 9,002-CO2), were calibrated before use.

Oxygen consumption (VO_₂_) and carbon dioxide production (VCO_₂_) were measured using an indirect calorimeter (KORR® Cardiocoach Plus, model 9002-CO_₂_), which employs breath-by-breath technology. Data were recorded every 15 s and then averaged on a minute-by-minute basis for subsequent analyses to ensure steady-state conditions.

### 2.5 Sample size

The sample size (*n* = 15 runners of each sex) was calculated using the equation proposed for clinical trials (32). An estimated effect size of d = 0.81, corresponding to a moderate difference in VO_2_ pre and post-supplementation (27), was used, with a significance level of *α* = 0.05 and a power of 90% (1 − *β* = 0.91). Based on these parameters, it was estimated that at least 21 participants were needed to detect significant differences between pre and post-intervention measurements. A power of 90% was selected to reduce the risk of a type II error due to the limited number of clinical trials available and the scarce existing evidence on glycerol supplementation. Ultimately, 30 competitive long-distance runners (15 men and 15 women) participated in the study.

### 2.6 Statistical methods

The Shapiro–Wilk test for paired samples was applied to evaluate the variables’ behavior within the dataset. On the basis of the distribution of the data, descriptive statistics were reported differently: for variables that followed a normal distribution, the mean and standard deviation (SD) were presented, whereas for variables with a nonnormal distribution, the median and interquartile range (IQR) were used. Additionally, an interaction analysis was conducted to examine whether sex modulated the response to glycerol supplementation. No significant interaction effect was found (*p* > 0.05), indicating that the responses were similar between male and female participants.

Differences between the glycerol supplementation (Gly) and control (No Gly) conditions were assessed through paired comparisons. For variables with a normal distribution, the paired t test was applied, whereas for nonnormally distributed variables, such as the OCU variable, the Wilcoxon signed-rank test for paired samples was used.

Additionally, 95% confidence intervals (CIs) for the mean differences were calculated for all normally distributed variables. For nonnormally distributed variables, such as the oxygen cost unit (OCU), the bootstrap method with accelerated bias correction (BCa) was used to calculate 95% BCa confidence intervals. A total of 1,000 replications were performed for each variable to obtain more representative confidence intervals, highlighting the specific characteristics of the data distribution.

For normally distributed variables, the effect size was calculated via Cohen’s d, whereas for the nonnormal OCU variable, the Wilcoxon effect size (*r*) was calculated. The interpretation of both effect sizes was based on the following thresholds: small (0.2), moderate (0.5), and large (0.8) for Cohen’s d and small (0.1), moderate (0.3), and large (0.5) for Wilcoxon’s r.

The statistical analysis was performed via R Studio version 4.4.2, with a confidence level of 95% and a statistical power of 90%, as specified in the sample size calculation. Statistical significance was considered for *p* values < 0.05.

## 3 Results

Thirty trained long-distance runners (15 males and 15 females) from various athletics clubs in Morelia, Michoacán, Mexico, were evaluated. The median age of the athletes was 27.0 years, the median peak oxygen consumption (VO_2_peak) was 62.09 mL·kg^−1^·min^−1^, and the median 10 km race time was 41:08:00 min. These and other key participant characteristics are detailed in Table 1.

**Table 1:** Participant characteristics.

Variables		Min	Max	Mean	SD
Age (y)		18.0	35.0	27.3	5.5
^**^Body Mass (kg)	No-Gly	43.2	74.9	60.0	8.6
	Gly ↑	43.8	76.6	61.4	8.9
Stretch stature (cm)		148.0	182.0	164.1	8.9
Rest Hearth Rate (bpm)		39.0	74.0	55.5	7.9
Hearth Rate Peak—P.T (bpm)		160.0	205.0	183.7	11.4
Maximal velocity—P.T (mph)		14.0	19.3	16.7	1.7
VO_2peak_—P.T (mL·kg^−1^·min^−1^)		47.6	79.3	62.1	7.4
10 k time (min)		38:07:00	54:00:00	42:08:00	5.5

P.T., values obtained from the Pugh Test conducted in the first session; Min, minimum value; Max, maximum value; SD, standard deviation; VO_₂_peak, peak oxygen consumption recorded during the P.T. Values are presented as means and standard deviations. ↑ Indicates an increase compared to its paired variable after glycerol supplementation. ^**^Indicates a significant difference (*p* < 0.01).

Based on the results obtained regarding body mass fluctuations, a paired t-test was conducted to compare the No-Gly and Gly conditions. The mean difference between the conditions was 1.34 kg, with a *p* value < 0.001, indicating a statistically significant difference. Additionally, the effect size (Cohen’s d = −3.82) was extremely large, suggesting a highly significant change in body mass between the two conditions.

Table 2 presents the comparison between the No-Gly and Gly sessions in relation to urine-specific gravity (USG) and climatic conditions. The results revealed a notable difference in USG, with an average value of 1.020 in the No-Gly group and 1.005 in the Gly group (*p* < 0.01), indicating that glycerol supplementation led to a significant decrease in urine specific gravity. With respect to the climatic conditions, no significant differences were found between the groups in wet-bulb temperature (WBT), temperature (T°), or relative humidity (RH), although a slight trend toward slightly higher temperatures was observed in the Gly group. The temperature (T°) remained constant in both groups, with an average of 21.10°C, whereas the relative humidity (RH) slightly varied, with an average of 56% for No-Gly and 55% for Gly.

**Table 2:** Comparison of USG and climatic conditions between the No-Gly and Gly groups.

Variable		Mean	Min	Max	SD
USG^**^	No-Gly	1.020	1.018	1.022	0.002
	Gly ↑	1.005	1.004	1.006	0.001
WBT (°C)	No-Gly	17.20	16.40	18.02	0.81
	Gly	17.80	16.60	18.00	0.72
T (°C)	No-Gly	21.10	20.80	21.30	0.26
	Gly	21.10	20.50	22.00	0.64
RH (%)	No-Gly	56.00	51.00	60.00	3.54
	Gly	55.00	50.50	58.50	3.39

Values are presented as means and standard deviations. USG, Urine Specific Gravity; WBT, Wet-Bulb Temperature (°C); T, Temperature (°C); RH, Relative Humidity (%). ↑ Indicates an increase compared to its paired variable after glycerol supplementation. ^**^Indicates a significant difference (*p* < 0.01).

Table 3 presents the results of the comparisons between the glycerol supplementation condition (Gly) and the nonglycerol condition (No-Gly) in five key variables: caloric cost unit (CCU), oxygen cost unit (OCU), heart rate (HR), rating of perceived excretion (RPE), and body temperature (BT), which are based on the average values from minutes 2–14 of each test.

**Table 3:** Comparison of running economy and performance variables with and without glycerol (*n* = 30).

Variable	No-Gly		Gly		Delta						*p*	*t*-statistic	Effect size
							95% CI No-Gly		95% CI Gly				
	Mean	SD	Mean	SD	ABS	%	Lower	Upper	Lower	Upper			Cohen’s *d*
CCU	1.13	0.11	1.11	0.11	0.02	2.25	1.09	1.17	1.07	1.15	0.025^*^	2.36	0.43
HR	146.32	12.86	143.60	15.00	2.71	2.14	141.51	151.12	138.00	149.20	0.029^*^	2.30	0.42
BT	36.75	0.26	36.67	0.22	0.08	0.21	36.65	36.85	36.59	36.75	0.053	2.02	0.37
RPE	9.51	1.17	8.52	1.81	0.98	18.22	9.07	9.94	7.85	9.20	0.003^*^	3.30	0.60

	Median	IQR	Median	IQR	ABS	%	95% BCa CI		95% BCa CI		*p*	Wilcoxon *r*
							Lower	Upper	Lower	Upper		
OCU	235.29	34.43	230.08	22.04	5.21	0.02	228.10	246.00	223.09	239.30	0.011^*^	0.46

CCU, Caloric Cost Unit (Kcal × kg^−1^ × km^−1^); HR, Heart Rate (bpm); BT, Body Temperature (°C); RPE, Rate of Perceived Exertion (6–20); OCU, Oxygen Cost Unit (mL × kg^−1^ × km^−1^); No-Gly, No Glycerol; Gly, Glycerol; CI, Confidence Interval; IQR, Interquartile Range; BCa, Bias-Corrected and Accelerated; ABS, Absolute Difference; %, Percentage Difference. The values for the CCU, HR, BT, RPE variables are presented as means and standard deviations, and were analyzed using the paired t test. The values for the OCU variable are presented as medians and interquartile ranges (IQR) and were analyzed using the Wilcoxon signed-rank test for paired data. Data were collected and analyzed from minutes 2 to 14 of the test. For body temperature (BT), measurements were taken at four specific time points during each session: before the test began, at minute 5, at minute 10, and at the end of the test (minute 15). ^*^Indicates a significant difference (*p* < 0.05).

The results indicate that glycerol supplementation had significant effects on most of these variables. In the RPE (Rating of Perceived Exertion), a significant difference was observed between the glycerol and nonglycerol conditions (*p* = 0.003). The mean RPE was 9.51 ± 1.17 before supplementation and 8.52 ± 1.81 after supplementation, with a mean difference of 0.98 (18.22%), suggesting considerable improvement in perceived exertion with glycerol. The effect size (Cohen’s d) was 0.60, indicating a large effect.

A significant difference in caloric cost units (CCUs) was found (*p* = 0.025), with a reduction in caloric cost under glycerol conditions. The mean CCU was 1.13 ± 0.11 before supplementation and 1.11 ± 0.11 after supplementation, with a mean difference of 0.02 (2.25%). The effect size was 0.43, suggesting a moderate effect.

Heart rate (HR) also significantly differed between the No-Gly and Gly conditions (*p* = 0.029), with a slightly lower frequency in the glycerol condition. The mean HR was 146.32 ± 12.86 in the No-Gly condition and 143.60 ± 15.00 in the Gly condition, with a mean difference of 2.71 (2.14%).

A slight reduction in body temperature (BT) was observed under glycerol conditions, although the difference was not statistically significant (*p* = 0.053). The mean BT was 36.75 ± 0.26 under the No-Gly condition and 36.67 ± 0.22 under the Gly condition, with a mean difference of 0.08, suggesting a slight trend toward a decrease in temperature but not reaching the significance threshold.

Finally, in the oxygen cost unit (OCU), a significant difference was found (*p* = 0.011), indicating an improvement in oxygen consumption efficiency after glycerol supplementation. The median difference was 5.21 (0.02%), with a moderate effect size (Wilcoxon r = 0.46).

**Figure 2:**
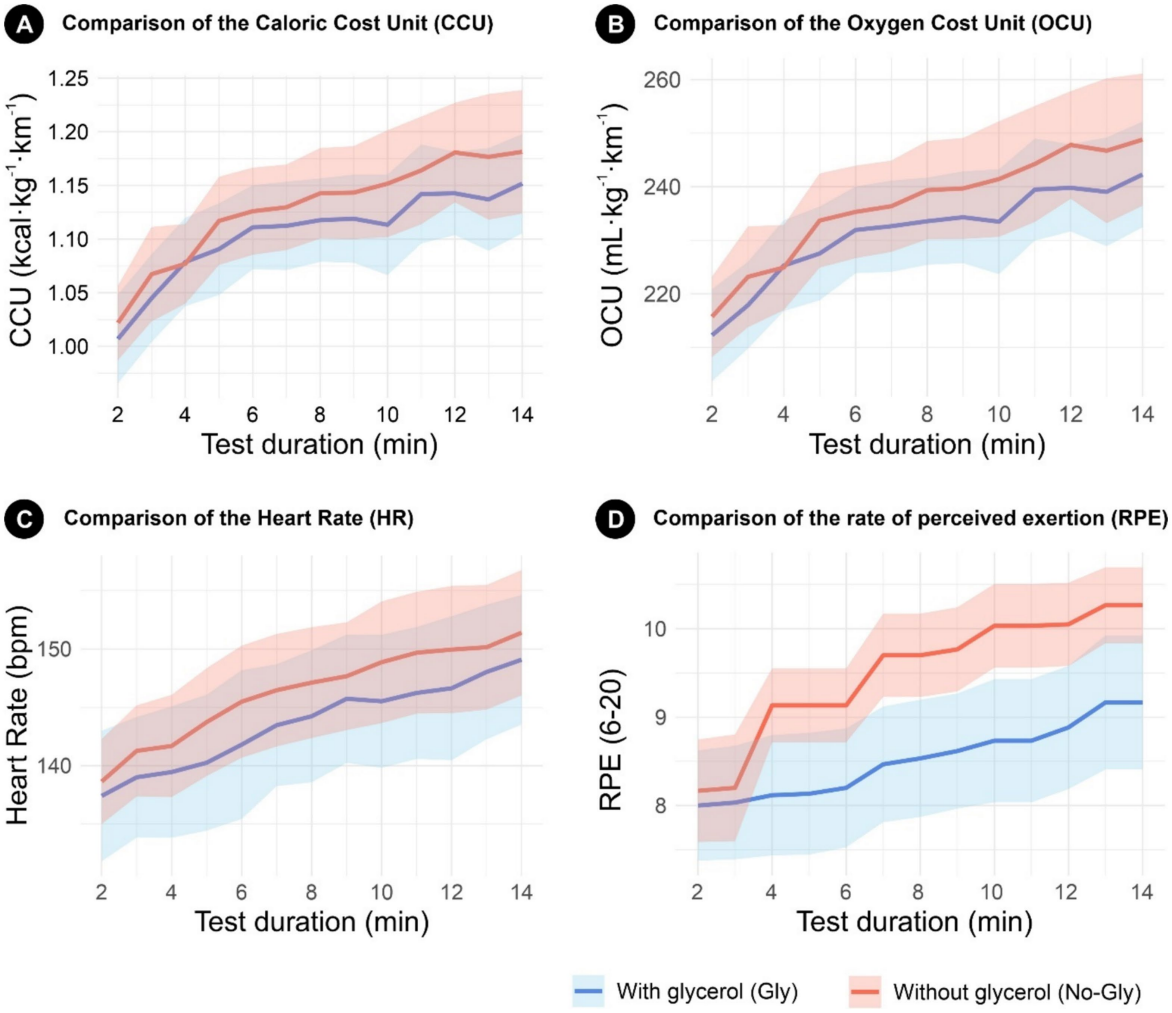
Comparison of the different variables with 95% confidence intervals (CIs). Solid lines represent the mean values for all variables except for the Oxygen Cost Unit (OCU, panel **B**), where the median is shown due to its non-normal distribution. For normally distributed variables, CIs were calculated for the mean differences; for OCU, 95% bootstrap confidence intervals (BCa method) were used. The graphs display the behavior of the variables from minute 2 to minute 14 of each trial. Statistical differences reflect global paired comparisons between the glycerol (Gly) and nonglycerol (No-Gly) conditions. CCU (panel **A**), Caloric Cost Unit (Kcal × kg^−1^ × km^−1^); OCU (panel **B**), Oxygen Cost Unit (mL × kg^−1^ × km^−1^); HR (panel **C**), Heart Rate (bpm); RPE (panel **D**), Rate of Perceived Exertion (6–20).

Figures 2A–D present the comparison of four key variables—caloric cost unit (CCU), oxygen cost unit (OCU), heart rate (HR), and rate of perceived exertion (RPE)—between the glycerol and nonglycerol conditions during the endurance treadmill test. The solid lines represent the mean values (or the median for OCU due to its non-normal distribution), and the shaded areas indicate the corresponding 95% confidence intervals (CIs). For OCU, 95% CIs were calculated using the bootstrap BCa method to account for its distribution characteristics. The statistical differences reported correspond to the global paired comparisons between conditions (paired t-test for normally distributed variables and Wilcoxon signed-rank test for nonnormally distributed variables), as reported in previous tables.

In Figure 2A, which shows the CCU, the nonglycerol group presented consistently higher values than did the glycerol group, with an average value of 1.13 kcal·kg^−1^·km^−1^ (95% CI: 1.09 to 1.17), whereas the value was 1.11 kcal·kg^−1^·km^−1^ (95% CI: 1.07 to 1.15) in the glycerol group.

In Figure 2B, which represents the OCU, the values were greater in the nonglycerol group, with a median of 235.29 mL·kg^−1^·km^−1^ (95% CI: 228.10 to 246.00), than in the glycerol group, at 230.08 mL·kg^−1^·km^−1^ (95% CI: 223.09 to 239.30).

In Figure 2C, The HR was greater in the nonglycerol group, with an average value of 146.32 bpm (95% CI: 141.51 to 151.12), than in the glycerol group, with a value of 143.60 bpm (95% CI: 138.00 to 149.20).

Finally, in Figure 2D, the rate of perceived exertion (RPE) was greater in the nonglycerol group, with an average value of 9.51 (95% CI: 9.07 to 9.94), than in the glycerol group, with an average value of 8.52 (95% CI: 7.85 to 9.20).

In all the graphs, the glycerol group presented narrower confidence intervals, indicating lower variability and greater consistency in the physiological response than the nonglycerol group did.

In summary, glycerol supplementation had a significant positive effect on perceived exertion, caloric cost, and oxygen efficiency, whereas heart rate was also affected, although to a lesser extent. Body temperature, on the other hand, did not significantly differ.

## 4 Discussion

The main findings of this study indicate that glycerol hyperhydration improves running economy in trained runners, refuting our initial hypothesis. This hypothesis was partially supported by a previous study (33), which demonstrated that reductions in body mass are associated with a decrease in the metabolic cost of running. This aligns with the idea that any intervention capable of influencing fluid retention and body mass could negatively impact running economy.

However, contrary to this expectation, glycerol supplementation in our study not only did not hinder running economy but also led to significant improvements in key parameters, including caloric cost, oxygen cost, cardiovascular responses, and perceived exertion. These findings suggest that the physiological mechanisms underlying glycerol supplementation may counteract the potential disadvantages of increased body mass, likely by optimizing fluid distribution and possibly enhancing thermoregulatory efficiency during exercise.

These findings highlight the complexity of the relationships among body mass, fluid retention, metabolic cost, and running performance. While previous research (33) provides a solid biomechanical explanation regarding the impact of body mass on energy expenditure, our results suggest that glycerol hyperhydration might overcome some of these limitations, likely because of its osmoregulatory effects and the improved hydration status it promotes (5, 6).

With respect to thermoregulatory responses, we observed a trend toward improved body temperature regulation, although the difference did not reach statistical significance. This observation was somewhat consistent with previous studies demonstrating that glycerol supplementation can have thermoregulatory effects on both body temperature (10, 27) and rectal temperature regulation (3, 11). Rectal temperature measurement is considered the most reliable technique for assessing changes in core body temperature. However, owing to its invasive nature, this approach was not feasible in this study. Instead, we used an infrared thermometer to measure body temperature. Although it was calibrated before use, it may have limited the accuracy of the assessment and the findings related to thermal regulation. Therefore, while the results suggest a potential thermoregulatory effect of glycerol, further studies with more precise methodologies are needed to confirm these findings and understand their magnitude in similar contexts.

Similarly, our results contrast with those reported by Beis et al. (27), who reported no significant effects of glycerol supplementation on running economy. Their study focused solely on raw VO_₂_ and VCO_₂_ data without considering variations in substrate utilization, which limits the depth of metabolic efficiency analysis. In contrast, our study incorporated more nuanced variables, such as unitary caloric cost (CCU) and unitary oxygen cost (OCU), allowing for a deeper and more precise evaluation of running economy. By integrating variables such as body mass and speed into the CCU and OCU equations, we were able to detect subtle differences in caloric and oxygen costs associated with supplementation. This more comprehensive approach provided a more detailed assessment of glycerol’s effects, revealing improvements in running economy that might not have been observed with a traditional analysis. We believe that if Beis et al. (27) employed a similar approach, they might have identified significant differences, emphasizing the importance of using tailored variables for a more accurate evaluation of running economy.

Martínez-Noguera et al. (34) also explored the effects of glycerol hyperhydration in elite racewalkers but did not observe significant improvements in running economy-related variables. Although their study included a placebo group, offering a methodological advantage over ours, the small sample size (only eight racewalkers) may have limited the statistical power and generalizability of their findings. In contrast, our study evaluated 30 trained runners, providing a robust sample size that was precalculated to ensure sufficient statistical power, reinforcing the reliability of our results.

Similarly, Knight et al. (18) examined the effect of glycerol ingestion on performance in multiple sport activities and reported a slight improvement in performance in the running segment after supplementation, but the overall difference was not statistically significant. Their study used a protocol of 1 g of glycerol per kilogram of body mass combined with 25 mL of fluid per kilogram of body mass, which was administered 30 min before exercise. Notably, studies reporting positive performance effects suggest that glycerol should be ingested between 120 and 180 min before exercise to maximize its benefits (3, 11, 17, 35–37). Furthermore, determining the optimal dosage is crucial for achieving significant benefits without adverse effects. The recommended ratio is 1.0 to 1.2 g of glycerol per kg of body mass combined with 20 to 25 mL of fluid per gram of glycerol (23, 24).

Although some studies have reported adverse effects associated with glycerol consumption—such as nausea, gastrointestinal discomfort, dizziness, and headaches—in a very small number of individuals (38, 39), none of the participants in the present study experienced any such symptoms. These findings suggest that the administered dose and ingestion protocol were well tolerated by our sample of trained runners.

In line with these findings, a recent study (40) reported no significant improvements in athletic performance despite enhanced hydration status following glycerol ingestion. However, our results provide a different perspective. Unlike that study, which only observed benefits in fluid retention, our findings not only show improvements in this aspect but also in key performance parameters, specifically in running economy. This contrast underscores the complexity of the effects of glycerol and highlights the importance of considering not only immediate physiological changes but also how these changes translate into actual impacts on athletic performance.

The differences between our findings and those of the most recent studies (27, 34, 40) evaluating glycerol’s impact on running performance could be related to glycerol’s positive effects on heart rate regulation (12, 36), a variable closely linked to metabolism, oxygen consumption, and energy expenditure (41–45). In this context, our findings support the hypothesis that the reduction in heart rate induced by glycerol supplementation is a key factor in improving running economy-related variables.

Overall, our results position glycerol hyperhydration as an effective and feasible strategy to improve running economy in trained runners. This study not only contributes to the current body of knowledge but also emphasizes the importance of designing personalized hydration strategies that optimize physiological responses during exercise. Future research should further explore the underlying mechanisms of these effects and assess the applicability of this strategy in specific sports disciplines and diverse environmental conditions, such as hot climates or prolonged competitions.

Nevertheless, some limitations must be acknowledged. First, the study did not employ a double-blinded design for the administration of glycerol and control beverages, which could have introduced some level of expectancy bias. Second, the duration of the running session was limited to 15 min. While sufficient to assess short-term responses, longer running durations might have revealed additional effects, particularly in terms of hydration status. Third, the laboratory conditions were thermoneutral; results may differ under heat stress, where hydration plays a more critical role. Fourth, although body temperature and hydration status were measured, the methods were limited in precision; future studies should incorporate more robust measures to strengthen data quality. Lastly, we did not measure plasma glycerol concentrations, which could have provided valuable insight into the absorption kinetics and physiological impact of the supplementation.

### 4.1 Practical applications

The results of this study suggest that glycerol-induced hyperhydration may be an effective and practical strategy to enhance running economy in trained athletes. This intervention can be easily implemented in endurance training or competition contexts, particularly in warm environments or prolonged efforts where thermoregulation and fluid balance are critical. Although glycerol supplementation resulted in a statistically significant increase in body mass (~1.4 kg on average), this did not impair running economy and therefore should not be a concern for athletes or practitioners considering this strategy. Nutritionists and sport practitioners may consider individualized pre-exercise hyperhydration protocols using glycerol to optimize performance while minimizing cardiovascular and metabolic strain. However, attention should be paid to proper dosage and timing, as well as the individual tolerance of athletes, to maximize benefits and reduce potential gastrointestinal discomfort or fluid overload.

## 5 Conclusion

This study demonstrated that glycerol hyperhydration significantly improves running economy, reduces cardiovascular strain, and lowers perceived exertion in trained runners under controlled conditions. While body temperature showed a trend toward improvement, this effect did not reach statistical significance, likely due to methodological limitations in its measurement. These findings highlight glycerol’s potential as a strategic tool to enhance endurance performance by mitigating physiological stress associated with prolonged exercise and suboptimal hydration. Future research should confirm these benefits in real-world conditions and assess their applicability across different athletic populations and environmental settings.

## Data Availability

The datasets presented in this study can be found in online repositories. The names of the repository/repositories and accession number(s) can be found at: https://figshare.com/articles/dataset/General_Data_Proyect_Glycerol_csv/28355384?file=52158935.
